# Improving Willingness to Try Fruits and Vegetables Among Low-Income Children Through Use of Characters

**DOI:** 10.1089/heq.2019.0113

**Published:** 2020-03-27

**Authors:** Caree J. Cotwright, Courtney Alvis, Fabiola de Jesus Jimenez, Paula Farmer, Chisom Okoli, Jennine Delane, Ginnefer O. Cox

**Affiliations:** ^1^ Department of Foods and Nutrition, College of Family and Consumer Sciences, University of Georgia, Athens, Georgia.; ^2^ Medical College of Georgia, Augusta University, Athens, Georgia.; ^3^ Clarke County School District Office of Nutrition, Athens, Georgia.

**Keywords:** characters, willingness to try, fruits and vegetables, low-income children, summer feeding, recipes, taste tests

## Abstract

**Purpose:** Low-income children are disproportionately affected by high rates of food insecurity and obesity, placing them at risk for poor health outcomes. Diets that are rich in fruits and vegetables (FV) are associated with health benefits such as reducing the risk of obesity. Despite these benefits, American children do not consume nationally recommended amounts of fruits (63%) and vegetables (90%) per day. Data reveal that young children exhibit increased food neophobia toward vegetables. One way to decrease food neophobia is to introduce FV to young children via recipe tasting. The purpose of the study was to increase willingness to try FV among low-income children using live characters at Summer Food Service Program Sites.

**Methods:** The study design was a small-scale pilot study to conduct taste tests of recipes with 125 low-income children. Researchers created recipe-tasting stations at two different sites. At Site 1, characters promoting FV characters were present at the recipe-tasting station. At Site 2, researchers presented recipes without characters being present. Taste tests were conducted once per week for a period of 4 weeks using two previously validated instruments, Taste Test Tool and the Taste and Rate Questionnaire.

**Results:** Results demonstrated that introducing FV recipes with characters showed a trend toward increasing willingness to try FV among low-income children. Data also suggested that low-income children had limited exposure to specific FV before recipe tastings.

**Conclusion:** The use of characters is a promising approach to increase willingness to try FV among low-income children.

## Introduction

The obesity epidemic affects children in the United States where 18.5% of children aged 2–19 years and 13.9% of young children aged 2–5 years are suffering from obesity.^1^ Specifically, in Georgia, approximately 30% of adolescent youth are overweight or obese.^2^ Children who are obese are more likely to continue to be obese as adults if corrective actions are not taken.^3,4^ Furthermore, childhood obesity is related to chronic illnesses such as hypertension, glucose intolerance, type 2 diabetes, and sleep apnea.^5^

Low-income children have disproportionately higher rates of obesity and food insecurity than those in the middle or upper class.^6^ In the United States, over 15 million children live in food insecure homes.^7^ Those who reside in low-income neighborhoods may have limited access to stores with healthy and affordable food, while being inundated with fast food options that are nutritionally poor and calorically dense.^8–10^ The combination of food insecurity and residing in an obesogenic environment can increase the risk of obesity for those who live in these low socioeconomic neighborhoods. Furthermore, poor eating habits influence early childhood obesity.^11^

Children's food preferences are shaped early in life and depend on the experiences and exposure they have had with food.^12,13^ Because children's food preferences are highly influenced by their exposure to different foods, it is vital to expose children to a wide variety of healthy foods early in life.^12–14^ Accordingly, as children age, it becomes more difficult to modify their food preferences.^12,14^ Therefore, early childhood is an ideal period to encourage and expose children to a variety of healthy foods, such as fruits and vegetables (FV). Increasing FV consumption among young children may reduce risk for obesity, chronic diseases, and establish healthy habits for adulthood.^15,16^

FV consumption is linked to a healthier childhood^17,18^ and adulthood.^19^ Diets that are rich in FV are associated with a multitude of health benefits such as a reducing the risk of obesity,^20^ cardiovascular disease,^21^ strokes,^22^ diabetes^23,24^ and certain cancers.^25,26^ According to the 2015–2020 Dietary Guidelines for Americans, children should consume between one and four cups of FV daily depending upon age.^27^ However, on average, the majority of children are not meeting their vegetable recommendations.^27^ One reason for children's lack of vegetable consumption is food neophobia. Food neophobia, or the fear of trying unfamiliar foods, is common in early childhood,^12,14^ thereby making food acceptance during these early years challenging. Food neophobia can be reduced by providing children with multiple exposures to the same types of foods.^13,14,28^ An increase in the number of times a child is exposed to a new food can result in the child liking the taste of this new food.^29^ Exploring novel approaches to introduce new foods, specifically, FV is important to increase FV consumption among children.

The use of characters has been found useful to influence taste preferences in children.^30^ For example, two separate studies found that children significantly preferred food products that had a fun character displayed on the package versus food that did not, although the food products were identical.^31,32^ In addition, characters from picture books and puppets have also been reported to influence children's food consumption and preferences.^33,34^ Results from a study where children were shown a puppet DVD for 5 consecutive days before lunch to influence FV consumption reported a significant increase in FV consumed from the test group compared to the control groups.^34^

The use of characters to influence children's food preferences in grounded in the social cognitive theory (SCT). According to SCT, developed by Albert Bandura, children learn through observational modeling by observing the behavior of those around them.^35^ Therefore, if a child sees a character, they admire doing a specific action (such as eating a healthy or nonhealthy food), and they may imitate this action and see themselves as being successful at completing the action.

Educating young children in using arts-based approaches results in increased understanding and a more positive attitude about the message.^36,37^ This approach may be promising for low-income children who lack exposure to FV. The goal of this study was to pilot the effectiveness of using characters to increase low-income children's willingness to try FV at recipe-tasting sites. This study focused on delivering FV taste test to children who participated in the Summer Food Service Program (SFSP) in Clarke County, GA. The United States Department of Agriculture (USDA) SFSP helps low-income children to continue to receive nutritious meals when school is out for summer vacation.^38^ Due to poverty levels in Clarke County, GA, the school district is a part of the Federal Community Eligibility Provision (CEP) from the USDA.^39^ The CEP allows free school meals to be served to each student without the basis of financial need.^39^ To implement CEP, at least 40% of students in the school district must qualify for free and reduced lunch based on National School Lunch Program guidelines.^39^ As the provision for school meals is highly important during the school year, it is important to find creative ways to increase consumption of healthy foods for low-income children during summer months.

Before this study, staff observation during SFSP meal delivery indicated that children frequently turned down healthy options included with meals such as FV (e.g., baby carrots). In response to this concern, researchers determined that providing novel exposure to FV at SFSP sites may be a promising way to increase willingness to try FV among low-income children. The goal of this study was to utilize the recipe taste test (RTT) to assess (1) children's willingness to try FV with and without the use of characters; (2) children's taste acceptability of selected FV recipes; and (3) children's willingness to try FV featured in recipes at home and at school. We hypothesized that low-income children would be more willing to try recipes presented with characters than without characters. We further hypothesized that low-income children would like the taste of recipes, and that low-income children would be willing to try the recipes again at home and at school.

## Methods

### Participants and recruitment

The study sample included 125 low-income children aged 2 to 18 who participated in the Clarke County School District (CCSD) SFSP sites (Table 1). Researchers worked with the CCSD school nutrition director to determine program sites. Parents were given a written assent letter and asked to give assent for children's participation and completion of a brief demographic survey. The University of Georgia Institutional Review Board approved all the study protocols before the start of data collection.

**Table 1.** Study Sample Characteristics of Children who Participated in Recipe Taste Test at Summer Food Service Program Sites in Clarke County, Georgia *N*=125

Characteristics	%
% African American	64
% Hispanic	24
% Caucasian	10.4
% Boys	46.4
% Girls	51.2

### Study design and measures

The study design was a small scale pilot study to conduct taste tests of FV recipes with low-income children. Over a 4-week period, participants were invited to take part in a voluntary RTT and told the activity was not related to receiving a summer meal. All recipes were tested first with a voluntary sample of participants and feedback was given to improve recipes. The study was conducted at two SFSP sites during the summer of 2016. At Site 1, characters promoting FV were present at the recipe-tasting station. The characters were called Freggie (fruits + veggies) and Friends. Freggie is a fun frog character that inspires healthy choices with his Green Machine (fruit and veggie cart). Freggie's Friends are life-sized FV characters. Each character represented the corresponding FV in the featured recipe (e.g., *Perri Pear*, *Cassie Carrot*), who were also present when recipe tastings were conducted. At Site 2, researchers presented the taste test at the tasting station without characters being present. One to two new recipes were introduced to participants each week. Each recipe-tasting station displayed the following: (1) a poster of the recipe ingredients; (2) specific information about the risk of allergies; (3) a list of symptoms of an allergic reaction; and (4) a care plan giving directions on what to do if an allergic reaction occurred. FV featured in recipes included pears, kiwi, pineapples, carrots, cauliflower, and sweet potatoes (Table 2).

**Table 2.** Recipes for Sample Taste Test with Low-Income Children at Summer Food Service Program Sites in Clarke County, Georgia

Fruit or vegetable for taste test	Recipe
Pear	Pear-adise Smoothie
Kiwi	Kiwi Pops
Sweet potato	Sweet Potato Bread
Carrot	Carrot Veggie Pockets
Pineapple	Pineapple Aloha Pasta
Cauliflower	Cauliflower Pop “Cauli”

Data were collected via The Taste Test Tool (TTT), and a Taste and Rate Questionnaire. The TTT, a brief seven-item survey, is a validated instrument^40^ used to evaluate food tasting activities among low-income children and adolescents. Researchers used the TTT to evaluate children's (1) previous exposure to the FV featured in the recipe, (2) whether or not they tried the recipe, and (3) whether or not they would try the recipe again at home and at school. The Site 1 TTT also included questions about whether or not children would be more likely to try FV in the presence Freggie characters. The Site 2 TTT did not address questions related to characters as characters were not present during the time of the taste test. The Taste and Rate Questionnaire created by Birch et al. was used to assess taste preferences for featured recipes.^41^ The tool includes a happy face scale for children to indicate how they like a recipe. The scale uses a smile, for “I really liked the recipe,” a blank face for “I sort of like the recipe,” and a frown for “I did not like the recipe.”^41^

Before participating in the recipe-tasting activity, children were told by a researcher that a study to learn more about how children like recipes with FV was taking place. Participants were told that they would taste a recipe and answer a brief survey. After tasting the recipe, the participant was administered the TTT. Participants were then asked to rate the taste of the recipe via a Taste and Rate Questionnaire. The survey was led by a graduate research assistant and took 5–10 min to complete. After completing the survey, participants were given a FV newsletter that contained the featured recipes from the tasting.

### Data analysis

Descriptive statistics and means were calculated using SPSS version 24.0 for the analysis of the exposure to FV, willingness to try FV, and taste preferences.

## Results

### Study sample

A total of 125 low-income children participated in the RTTs at SFSP Sites 1 and 2. Participants identified as African American (64%), Hispanic (24%), and Caucasian (10.4%). Fifty-one percent of the participants were girls.

### Recipe taste tests

Overall results for the RTTs are presented in Table 3 and Table 4. During week 1 (*Pear* RTT), all participants at both sites (Site 1: with Freggie [WF]; Site 2: No Freggie [NF]) had seen a pear before. In addition, all participants (*n*=11, 100%) exposed to Freggie liked the Pear recipe compared to those without Freggie (*n*=6, 67%). Eight children (73%) at Site 1 (WF) reported that they were willing to try the pear recipe due to the presence of Freggie, however, 10 participants (91%) reported that they would have still tried the pear recipe without Freggie being present.

**Table 3.** Children's Recipe Taste Test with Freggie and Friends Characters Present at Summer Feeding Site During Summer 2016

Questions	FV featured in taste test, N^a^=57											
	Pear		Kiwi		Sweet potato		Carrot		Pineapple		Cauliflower	
	n^b^	y, %Y	n^b^	y, %Y	n^b^	y, %Y	n^b^	y, %Y	n^b^	y, %Y	n^b^	y, %Y
Did you like the recipe?	11	11, 100	8	8, 100	10	7, 70	15	10, 67	6	3, 50	7	2, 29
Have you seen this FV before?	11	11, 100	8	6, 75	10	6, 60	15	15, 100	6	5, 83	7	5, 71
Have you tried this FV before?	11	10, 91	8	6, 75	10	6, 60	15	12, 80	6	3, 83	7	3, 43
Would you try this FV again at school?	11	9, 82	8	7, 88	10	8, 80	15	12, 80	6	3, 50	7	3, 43
Would you try this FV again at home?	11	9, 82	8	7, 88	10	8, 80	15	12, 80	6	3, 50	7	2, 29
Did you try this FV today because you saw Freggie?	11	8, 73	8	7, 88	10	10,100	15	13, 87	6	6, 100	7	6, 86
Would you have tried this FV without Freggie?	11	10, 91	8	6, 75	10	7, 70	15	7, 47	6	2, 33	7	1, 14

Summer Food Service Program Site 1 with Freggie and Friends characters. Freggie and Friends are fruit and vegetable characters that promote healthy eating.

^a^ Total number of participants at Site 1.

^b^ Number of participants who participated in the respective FV RTT.

y, Number of participants who responded “YES” to questions; %Y, Percentage of participants who responded “YES” to questions.

FV, fruit and vegetable; RTT, recipe taste testing.

**Table 4.** Children's Recipe Taste Test Without Freggie and Friends Characters Present at Summer Food Service Program Site in Clarke County, Georgia

	FV featured in taste test, N^a^=68											
	Pear		Kiwi		Sweet potato		Carrot		Pineapple		Cauliflower	
Questions	n^b^	y, %Y	n^b^	y, %Y	n^b^	y, %Y	n^b^	y, %Y	n^b^	y, %Y	n^b^	y, %Y
Did you like the recipe?	9	6, 67	5	5, 100	17	17,100	11	8, 73	10	9, 90	16	9, 57
Have you seen this FV before?	9	9, 100	5	5, 100	17	6, 35	11	11, 100	10	10, 100	16	9, 57
Have you tried this FV before?	9	7, 78	5	5, 100	17	6, 35	11	11, 100	10	10, 100	16	8, 50
Would you try this FV again at school?	9	8, 89	5	5, 100	17	17,100	11	7, 64	10	10, 100	16	9, 57
Would you try this FV again at home?	9	8, 89	5	5, 100	17	17,100	11	8, 73	10	10, 100	16	9, 57

Summer Food Service Program Site 2 with Freggie and Friends characters. Freggie and Friends are fruit and vegetable characters that promote healthy eating.

^a^ Total number of participants at Site 2.

^b^ Number of participants who participated in the respective FV RTT.

y, Number of participants who responded “YES” to questions; %Y, Percentage of participants who responded “YES” to questions.

During week 2 (*Kiwi* RTT), six out of eight participants (75%) at Site 1 (WF) and all participants (5, 100%) at Site 2 (NF) had previously seen a kiwi. All participants at both sites reported that they liked the Kiwi recipe. More children were willing to try the kiwi recipe because of the presence of Freggie (*n*=7, 88%), compared to six out of eight children (75%) who were willing to try the recipe without Freggie.

During week 3, there were two RTTs (*sweet potato* RTT and *carrot* RTT). For the sweet potato RTT, six children (*n*=10, 60%) at Site 1 and six children (*n*=17, 35%) at Site 2 had seen a sweet potato before. All participants (*n*=17) at Site 2 (NF) liked the recipe compared to 7 out of 10 participants (70%) at Site 1 (WF). At Site 1 (WF), all participants (*n*=10, 100%) were willing to try the sweet potato recipe with Freggie present compared to seven children who reported that they would try without Freggie's presence (*n*=10, 70%). For the carrot RTT, all participants at both sites had previously seen a carrot. Ten children (*n*=15, 67%) from Site 1 (WF) and eight children (*n*=11, 73%) from Site 2 (NF) liked the recipe. In addition, 13 children (*n*=15, 87%) were willing to try the carrot recipe as a result of Freggie's presence compared to seven children (*n*=15, 47%) who would try the recipe without Freggie at Site 1.

During week 4, two recipes were also tested (*Pineapple* RTT and *Cauliflower* RTT). In the pineapple RTT, five out of six participants (83%) at Site 1 (WF) and all participants (100%) at Site 2 (NF) had seen a pineapple before. After tasting, three children (*n*=6, 50%) at Site 1 and nine children (*n*=10, 90%) at Site 2 liked the pineapple recipe. At Site 1 (WF), children (*n*=6, 100%) were more willing to try the recipe due to the presence of Freggie compared to two children (*n*=6, 33%) who agreed that they would try the recipe without Freggie at Site 2 (NF). During the cauliflower RTT, 5 out of 7 children (71%) at Site 1 (WF) and 9 out of 16 children (57%) at Site 2 (NF) had previously seen a cauliflower. Nine participants (*n*=16, 57%) at Site 2 and two participants (*n*=7, 29%) at Site 1 liked the recipe. Six out of seven children at Site 1 (WF) (86%) were more than willing to try the cauliflower recipe due to the presence of Freggie compared with only one child who reported that he would try the recipe without Freggie (*n*=7, 14%).

## Discussion

The present investigation studied the influence of characters on low-income children's willingness to try FV. During our recipe tastings, five out of the six recipes displayed at Site 1 (WF) showed a trend of children improving willingness to try a recipe due to the presence of Freggie (character used) compared to children who would try without Freggie's presence. Our findings are consistent with previous studies that have found the influence of characters on children's willingness to try FV.^31,33^ For example, book characters in a book promoting carrots have also been successful at increasing children's carrot consumption.^31^ Further in a follow-up study, de Droog et al. further found that an interactive reading style using character imitation resulted in the highest carrot consumption.^33^

We also found that children enjoyed the fruit recipes more than the vegetable recipes. According to the Dietary Guidelines of 2015–2020, in the United States, 90% of children are not meeting their daily vegetable needs.^27^ Specifically, for children aged 1–18 years, vegetable recommendations are not met in any childhood life stage compared to fruit intake met at 1–8 years of age.^27^ Thus, data reveal that American children are less likely to eat vegetables than fruits.

In addition, our results revealed that recipes that resembled foods commonly eaten by children such as the sweet potatoes were better accepted than foods such as cauliflower. This finding is generally consistent with the scientific literature where children's willingness to try FV is highly dependent on their exposure to these foods at an early age.^41^ A study by Forestell and Mennella found that when infants were weaning from breast milk or formula, they were more likely to accept a new food product if they have had several opportunities to try it.^42^ Furthermore, a systematic review of the literature summarized that children's main influence for food preferences was the familiarity with the food.^14^

Finally, we found that low-income children who reported liking a recipe were much more likely to indicate that they would try the recipe again at school or at home, independent of the presence of Freggie and Friends characters. The practical relevance of this investigation was to utilize the recipes children reported they were willing to try again at school or at home for inclusion on school menus and new recipes for SFSP sites in Clarke County, Georgia. This is important to increase FV consumption among low-income children in the CCSD. Serving menu items that are proved to be healthy and liked by children is also important as this can help reduce food waste in schools.

This study was unique to investigating the impact of an entertaining healthy taste test intervention among low-income children aged 2–18 years. This investigation was particularly unique in using creative recipes such as the Pear-adise Smoothie that are more appealing to children than a bland uncooked food item. The present study, to our knowledge, was the first to test the impact of live characters in costume on children's willingness to try FV at summer feeding sites. Other strengths of the study included the poster displaying the recipe ingredients and allergy information.

The study also had some limitations. First is that this investigation was small-scale study with a sample size of 125 children from only Clarke County, Georgia. Having a small sample from only one region does not give an accurate representation of low-income children aged 2–18 years throughout Georgia. Nonetheless, this study is one of the firsts to investigate live characters in costume and their impact of children's willingness to try FV. A larger sample size is therefore needed to test the intervention. Second, our sample size included a very wide range of ages for children (2–18 years) and this could have affected our findings for character influence on children's willingness to try FV. Previous studies have found that young children are more influenced by characters than older children.^43^ Finally, researchers could only perform taste test with children who were present at summer feeding sites with legal guardians to provide assent. Therefore, participation in the study at Summer Feeding Program Sites varied highly from week to week during the study period.

### Health equity implications

This study provides a replicable model to promoting FV at SFSP sites in partnership with a large school district serving low-income children. As low-income children experience higher rates of food insecurity and obesity, increasing FV consumption may be a way to decrease chronic disease risk.^6^ In addition, collaborative projects with school systems may provide greater access to low-income children to promote FV at SFSP sites, at home, and at school. Future research should determine children's preferences for characters and children's input on specific FV to feature in recipes. Newly developed interventions should include a greater number of children and sites. Furthermore, future studies may consider enrolling children during the school year so that children can participate in recipe tasting when a legal guardian is not present.

## Abbreviations Used

CCSD: Clarke County School District
CEP: Community Eligibility Provision
FV: fruits and vegetables
NF: no Freggie
RTT: recipe taste test
SCT: social cognitive theory
SFSP: Summer Food Service Program
TTT: Taste Test Tool
USDA: United States Department of Agriculture
WF: with Freggie

## References

[1] State of Childhood Obesity; Robert Wood Johnson Foundation. National Obesity Monitor. 2019. Available at https://stateofchildhoodobesity.org/monitor Accessed 114, 2019

[2] Centers for Disease Control and Prevention. Georgia state nutrition, physical activity, and obesity profile. Available at https://www.cdc.gov/nccdphp/dnpao/state-local-programs/profiles/pdfs/georgia-state-profile.pdf (last accessed 317, 2020)

[3] Hill JO, Trowbridge FL Childhood obesity: future directions and research priorities. Pediatrics. 1998;101:570–574 12224663 10.1542/peds.101.3.570

[4] Charney E, Goodman HC, McBride M, et al. Childhood antecedents of adult obesity—do chubby infants become obese adults? N Engl J Med. 1976;295:6–9 1272299 10.1056/NEJM197607012950102

[5] Centers for Disease Control and Prevention. Childhood obesity causes and consequences. 2016 Available at https://www.cdc.gov/obesity/childhood/causes.html Accessed Novermber 4, 2019

[6] The State of Obesity. Better policies for a healthier America: socioeconomics and obesity. 2017. Available at http://stateofobesity.org/socioeconomics-obesity Accessed 114, 2019

[7] Coleman-Jensen A, Rabbitt MP, Gregory C, et al. Household Food Security in the United States in 2015. Washington, DC: U.S. Department of Agriculture, Economic Research Services, 2016

[8] Food Research & Action Center. Understanding the connections: food insecurity and obesity. 2015. Available at http://frac.org/wp-content/uploads/frac_brief_understanding_the_connections.pdf Accessed 114, 2019

[9] Treuhaft S, Karpyn A The grocery gap: who has access to healthy food and why it matters. Available at http://thefoodtrust.org/uploads/media_items/grocerygap.original.pdf Accessed 114, 2019

[10] Bell J, Mora G, Hagan E, et al. Access to healthy food and why it matters: a review of the research. Available at http://thefoodtrust.org/uploads/media_items/executive-summary-access-to-healthy-food-and-why-it-matters.original.pdf Accessed 114, 2019

[11] Campbell K, Hesketh K, Crawford D, et al. The infant feeding activity and nutrition trial (infant) an early intervention to prevent childhood obesity: cluster-randomized controlled trial. BMC Public Health. 2008;8:103 18373877 10.1186/1471-2458-8-103 PMC2346474

[12] Aldridge V, Dovey TM, Halford JC The role of familiarity in dietary development. Dev Rev. 2009;29:32–44

[13] Dazeley P, Houston-Price C, Hill C Should healthy eating programmes incorporate interaction with foods in different sensory modalities? A review of the evidence. Br J Nutr. 2012;108:769–777 22264626 10.1017/S0007114511007343

[14] Cooke L The importance of exposure for healthy eating in childhood: a review. J Human Nutr Diet. 2007;20:294–301 17635306 10.1111/j.1365-277X.2007.00804.x

[15] Lau RR, Quadrel MJ, Hartman KA Development and change of young adults' preventive health beliefs and behavior: influence from parents and peers. J Health Soc Behav. 1990;240–259 2133479

[16] Wardle J Parental influences on children's diets. Proc Nutr Soc. 1995;54:747–758 8643712 10.1079/pns19950074

[17] Lin BH, Morrison RM Higher fruit consumption linked with lower body mass index. Food Rev. 2002;25:28–32

[18] Tohill BC Dietary Intake of Fruit and Vegetables and Management of Body Weight. Geneva, Switzerland: World Health Organization (WHO), 2005

[19] De Kroon ML, Renders CM, Van Wouwe JP, et al. The Terneuzen birth cohort: BMI changes between 2 and 6 years correlate strongest with adult overweight. PLoS One. 2010;5:e9155 20161800 10.1371/journal.pone.0009155 PMC2820098

[20] McCrory MA, Fuss PJ, McCallum JE, et al. Dietary variety within food groups: association with energy intake and body fatness in men and women. Am J Clin Nutr. 1999;69:440–447 10075328 10.1093/ajcn/69.3.440

[21] Joshipura KJ, Hu FB, Manson JE, et al. The effect of fruit and vegetable intake on risk for coronary heart disease. Ann Intern Med. 2001;134:1106–1114 11412050 10.7326/0003-4819-134-12-200106190-00010

[22] Joshipura KJ, Ascherio A, Manson JE, et al. Fruit and vegetable intake in relation to risk of ischemic stroke. JAMA. 1999;282:1233–1239 10517425 10.1001/jama.282.13.1233

[23] Cooper AJ, Sharp SJ, Luben RN, et al. The association between a biomarker score for fruit and vegetable intake and incident type 2 diabetes: the EPIC-Norfolk study. Eur J Clin Nutr. 2015;69:449–454 25387899 10.1038/ejcn.2014.246 PMC4704139

[24] Ford ES, Mokdad AH Fruit and vegetable consumption and diabetes mellitus incidence among US adults. Prev Med. 2001;32:33–39 11162324 10.1006/pmed.2000.0772

[25] Steinmetz KA, Potter JD Vegetables, fruit, and cancer prevention: a review. J Am Diet Assoc. 1996;96:1027–1039 8841165 10.1016/S0002-8223(96)00273-8

[26] Willet WC, Trichopoulos D Nutrition and cancer: a summary of the evidence. Cancer Causes Control. 1996;7:178–180 8850444 10.1007/BF00115648

[27] United States Department of Agriculture, United States Department of Health and Human Services Dietary Guidelines for Americans 2015-2020. 2015. Available at https://health.gov/dietaryguidelines/2015/resources/2015-2020_Dietary_Guidelines.pdf Accessed 114, 2019

[28] Birch LL, Davison KK Family environmental factors influencing the developing behavioral controls of food intake and childhood overweight. Pediatr Clin. 2001;48:893–907 10.1016/s0031-3955(05)70347-3 11494642

[29] Anzman-Frasca S, Savage JS, Marini ME, et al. Repeated exposure and associative conditioning promote preschool children's liking of vegetables. Appetite. 2012;58:543–553 22120062 10.1016/j.appet.2011.11.012

[30] Roberto CA, Baik J, Harris JL, et al. Influence of licensed characters on children's taste and snack preferences. Pediatrics. 2010;126:88–93 20566614 10.1542/peds.2009-3433

[31] de Droog SM, Buijzen M, Valkenburg PM Enhancing children's vegetable consumption using vegetable-promoting picture books: the impact of interactive shared reading and character–product congruence. Appetite. 2014;73:73–80 24216486 10.1016/j.appet.2013.10.018

[32] Enax L, Weber B, Ahlers M, et al. Food packaging cues influence taste perception and increase effort provision for a recommended snack product in children. Front Psychol. 2015;6:882 26191012 10.3389/fpsyg.2015.00882 PMC4488606

[33] de Droog SM, van Nee R, Govers M, et al. Promoting toddlers' vegetable consumption through interactive reading and puppetry. Appetite. 2017;116:75–81 28438548 10.1016/j.appet.2017.04.022

[34] Nicklas T, Lopez S, Liu Y, et al. Motivational theater to increase consumption of vegetable dishes by preschool children. Int J Behav Nutr Phys Act. 2017;14:16 28166788 10.1186/s12966-017-0468-0 PMC5294896

[35] Bandura A Human agency in social cognitive theory. Annu Rev Psychol. 1989;44:1175–1184 10.1037/0003-066x.44.9.1175 2782727

[36] Buijzen M, Van Reijmersdal EA, Owen LH Introducing the PCMC model: an investigative framework for young people's processing of commercialized media content. Commun Theory. 2010;20:427–450

[37] Fisch SM A capacity model of children's comprehension of educational content on television. Media Psychol. 2000;2:63–91

[38] United States Department of Agriculture. Summer Food Service Program. 2019. Available at https://www.fns.usda.gov/sfsp/summer-food-service-program Accessed 114, 2019

[39] United States Department of Agriculture; The Community Eligibility Provision (CEP). What does it mean for your school or local educational agency? 2015. Available at https://fns-prod.azureedge.net/sites/default/files/cn/CEPfactsheet.pdf Accessed 114, 2019

[40] Kaiser LL, Schneider C, Mendoza C, et al. Development and use of an evaluation tool for taste-testing activities by school-aged children. J Acad Nutr Diet. 2012;112:2028–2034 23063553 10.1016/j.jand.2012.07.006

[41] Birch LL, Zimmerman SI, Hind H The influence of social-affective context on Preschool Children's Food Preferences. Child Dev. 1980;51:856–861

[42] Forestell CA, Mennella JA Early determinants of fruit and vegetable acceptance. Pediatrics. 2007;120:1247–1254 18055673 10.1542/peds.2007-0858 PMC2268898

[43] Ogle AD, Graham DJ, Lucas-Thompson RG, et al. Influence of cartoon media characters on children's attention to and preference for food and beverage products. J Acad Nutr Diet. 2017;117:265–270 27793520 10.1016/j.jand.2016.08.012 PMC5574189

